# Type I interferon signaling restrains IL-10R^+^ colonic macrophages and dendritic cells and leads to more severe *Salmonella* colitis

**DOI:** 10.1371/journal.pone.0188600

**Published:** 2017-11-30

**Authors:** Kailyn L. Stefan, Avner Fink, Neeraj K. Surana, Dennis L. Kasper, Suryasarathi Dasgupta

**Affiliations:** 1 Department of Microbiology and Immunobiology, Harvard Medical School, Boston, Massachusetts, United States of America; 2 Division of Infectious Diseases, Department of Medicine, Boston Children’s Hospital, Boston, Massachusetts, United States of America; Kurume University School of Medicine, JAPAN

## Abstract

Type I interferons (IFNα, IFNβ) are key regulators of innate and adaptive immunity, modulating the severity of both viral and bacterial infections. While type I IFN signaling leads to improved outcomes in viral infections, its role in bacterial infections is more contextual and depends on the specific pathogen and route of infection. Given the limited evidence on whether type I IFN signaling affects enteric bacterial pathogens, we investigated the role of this signaling pathway in *Salmonella enterica* serovar Typhimurium (*S*. *typhimurium*)–induced colitis. Comparing mice deficient in IFNAR1- the common receptor for IFNα and IFNβ- with wild-type mice, we found that type I IFN signaling leads to more rapid death, more severe colonic inflammation, higher serum levels of pro-inflammatory cytokines, and greater bacterial dissemination. Specific ablation of plasmacytoid dendritic cells (pDCs), which are prominent producers of type I IFNs in antiviral responses, did not alter survival after infection. This result established that pDCs do not play a major role in the pathogenesis of *S*. *typhimurium* colitis. Flow cytometric analysis of macrophages and conventional dendritic cells (cDCs) during active colitis demonstrated an increase in CD11c^-^ macrophages and CD103^+^ cDCs in the colon of *Ifnar1*^*-/-*^ animals. Interestingly, cells expressing the anti-inflammatory cytokine receptor IL-10R are more abundant within these subsets in *Ifnar1*^*-/-*^ than in wild-type mice. Moreover, blockade of IL-10R in *Ifnar1*^*-/-*^ mice increased their susceptibility to *S*. *typhimurium* colitis, suggesting that altered numbers of these immunoregulatory cells may underlie the difference in disease severity. This cross-talk between type I IFN and IL-10R signaling pathways may represent a key host cellular mechanism to investigate further in order to unravel the balance between pathogenic inflammation and homeostasis of the colon. Taken together, our data clearly demonstrate that type I IFN signaling is pathogenic in *S*. *typhimurium* colitis.

## Introduction

Pathogen-induced inflammation is a natural response that is beneficial to the host as an initial mechanism of defense. However, excessive inflammation caused by an overwhelming immune response can lead to severe damage to the host, including organ dysfunction, septic shock, and death [1]. Thus, the balance of the inflammatory response must be tightly regulated to avoid excessive damage to the host. Type I interferon (IFN) encompasses a class of cytokines that play a key role in the balance between immunity and pathogenesis. Type I IFN’s causal role in pathology has been demonstrated in the setting of various autoimmune diseases, such as lupus and multiple sclerosis [2, 3]. Both of the major type I IFNs, α and β, signal through a common receptor, IFNAR1. Studies in mice deficient in IFNAR1 have elucidated both the immunity-enhancing and pathogenic roles of type I IFN.

The role of type I IFNs in modulating infection has been most extensively studied in the context of viral infections, in which they promote immunity. While there is burgeoning evidence that type I IFNs also modulate the severity of bacterial infections, this effect appears to depend on context [4]. For example, type I IFNs are protective against infections with many extracellular bacteria (e.g., *Streptococcus* species, *Pseudomonas aeruginosa*) but are detrimental during infection with intracellular bacteria (e.g., *Francisella tularensis*, *Brucella abortus*) [5, 6]. Moreover, the route of infection is important: in mice, type I IFN signaling during *Listeria* infections is pathogenic in parenteral infections but beneficial in enteric ones, the latter mimicking the natural route of infection for this pathogen in humans [7]. An important proof-of-concept study recently demonstrated that type I IFN signaling is detrimental in *Salmonella* infection; however, this study used non-physiologic routes of infection- i.e., intravenous and intraperitoneal injection [8]. This study showed that during *Salmonella enterica* serovar Typhimurium (*S*. *typhimurium*) infection *in vitro*, IFN signaling induces necroptosis in infected macrophages; therefore, *Ifnar1*^*-/-*^ macrophages are resistant to *S*. *typhimurium*-induced cell death. Murine infections with *S*. *typhimurium* can model either a typhoid-like illness with prominent systemic dissemination (with no antibiotic pre-treatment) or primarily gastroenteritis combined with systemic dissemination (after pre-treatment with streptomycin) [9]. This facultative intracellular bacterium causes an estimated 90 million cases of gastroenteritis per year [10], displaying an intricate interplay with the human immune system [11]. Using the typhoid model of *Salmonella* infection, investigators have recently shown that IFNβ promotes bacterial dissemination- an observation consistent with the idea that type I IFN signaling is detrimental under these conditions [12]. Given the discordant results for systemic and enteric infections with *Listeria monocytogenes*, we wanted to determine what role type I IFN signaling plays in *Salmonella*-induced colitis. Moreover, we wanted to explore the relevant immune-cell subsets involved in type I IFN signaling during *Salmonella* infection, about which only few details are known.

In this study, we found that type I IFN signaling is pathogenic in *S*. *typhimurium*-induced colitis, with wild-type (WT) mice dying faster than *Ifnar1*^*-/-*^ mice. This greater susceptibility to mortality in WT mice was associated with increased dissemination of bacteria, increased serum levels of pro-inflammatory cytokines, and increased colonic inflammation. Numbers of CD11c^-^ colonic macrophages, which may be similar to CD11c^lo^ macrophages that reside in the muscularis mucosa region and regulate gut motility [13], as well as numbers of CD103^+^ dendritic cells, which play an important role in inflammatory diseases and antigen carriage from intestine to mesenteric lymph nodes (MLNs) [14], were higher in infected *Ifnar1*^*-/-*^ mice than in infected WT mice. Notably, these cell types expressing the immunoregulatory interleukin 10 receptor (IL-10R) were similarly increased in *Ifnar1*^*-/-*^ mice. These results suggest that type I IFN signaling leads to a loss of these cell types that is associated with worse overall outcomes. Indeed, blockade of IL-10R in *Ifnar1*^*-/-*^ mice resulted in increased susceptibility to *S*. *typhimurium* colitis, eliminating the difference in mortality compared to WT mice. Thus, type I IFN signaling is pathogenic in *Salmonella* colitis, and its pathogenicity is likely to be a result of decreased numbers of immunoregulatory macrophages and dendritic cells.

## Material and methods

### Mice

*Ifnar1*^*-/-*^ mice (B6.129S2-*Ifnar1tm1Agt*/Mmjax, stock number 010830) and BDCA2-DTR mice (C57BL/6-Tg(CLEC4C-HBEGF)956Cln/J, stock number 014176) on a C57BL/6 background were purchased from Jackson Laboratory (Bar Harbor, ME). The genotype of C57BL/6-Tg(CLEC4C-HBEGF)956Cln/J mice was confirmed by polymerase chain reaction with a previously described protocol [15]. All genetically deficient mice and their respective controls were age-matched (6–8 weeks) and were co-housed under specific pathogen free (SPF) conditions. All experiments on animals were approved by the Harvard Medical Area Standing Committee on Animals (animal protocol number IS00000187).

### *S*. *typhimurium*-induced infectious colitis

Streptomycin sulfate in PBS (20 mg/mouse, Sigma-Aldrich) was administered to mice via oral gavage. At 24 hours after treatment, 10^2^ to 10^7^ CFU of *S*. *typhimurium* strain SL1344 (grown overnight at 37°C in LB Broth, Miller (Luria-Bertoni) with added streptomycin, washed and diluted in PBS) was administered to each mouse via oral gavage. A dose of 10^4^ CFU was selected following dose response studies. For survival studies, body mass was measured daily and mice were observed for symptoms of pain or distress as per our institutional animal protocol until the end of the experiment. Mice were euthanized immediately and reported as dead once body condition score of less than 2 out of 5 was observed or they appeared moribund. Approximately less than 5% of animals were found dead beyond supervision and were considered to have died on the day of observation for the purpose of data analysis. For flow cytometric analysis, serum cytokine quantification, bacterial burden assessment, and histopathology studies, mice were euthanized on days 1, 3 or 5 after infection.

### Cell isolation from primary tissues

For single-cell suspensions of mesenteric lymph nodes (MLNs), the first five MLNs (starting from the cecum) were treated with collagenase type IV (1 mg/ml; Sigma) for 30 minutes at 37°C in an atmosphere of 5% CO_2_ and passed through a 70-μm mesh. Single cells were isolated from colonic tissue as previously described [15]. In brief, the tissue was first cut transversely into small pieces and then cut longitudinally to expose the lumen. Pieces were washed in 1 mM dithiothreitol (Sigma) in PBS for 10 minutes and then in PBS. Three washes of 8 minutes each in 30 mM EDTA in PBS were then employed to strip off epithelial cells. After one more wash in PBS, tissue was treated with RPMI medium containing 5% fetal bovine serum (FBS) and collagenase type IV (1 mg/ml) for 1 hour at 37°C in 5% CO_2_. Finally, tissue sections were passed through mesh (70-μm) to yield single-cell suspensions.

### Flow cytometric analysis

The following mouse-specific IgG monoclonal antibodies were purchased from BioLegend (San Diego): biotin-conjugated anti-MHCII; Brilliant Violet 605-conjugated streptavidin; Pacific Blue–conjugated anti-CD45; allophycocyanin-conjugated anti-F4/80; fluorescein isothiocyanate-conjugated anti-B220; phycoerythrin-conjugated anti-IL-10R and anti-SH; PerCP-Cy5.5-conjugated anti-CD11b; PE-Cy7-conjugated anti-CD11c; and Brilliant Violet 510-conjugated anti-CD103. Fixable Viability Dye eFluor780 was purchased from eBioscience (San Diego). Single-cell suspensions from primary tissues were stained with a suitable combination of fluorochrome-conjugated antibodies and Fixable Viability Dye, fixed in 2% paraformaldehyde in PBS, and examined with a BD LSR II flow cytometer (Becton, Dickinson). The data were analyzed with FlowJo software. For counting of cells by flow cytometry, Flow-Count Fluorospheres (Beckman Coulter, Brea, CA) were used per instructions provided by the manufacturer.

### Serum cytokine quantification

Mice were treated with streptomycin and infected with 10^4^ CFU of *S*. *typhimurium* strain SL1344 as described above. On day 5, mice were euthanized, and blood was collected into 1.1-ml Z-Gel Micro Tubes (Sarstedt). Blood was left at room temperature for 30 minutes before the coagulated cells were spun out (10,000 *g*, 10 minutes), and serum was stored at -80°C. Levels of tumor necrosis factor α (TNFα) and interleukin-6 (IL-6) were quantified with ELISA Duoset kits (R&D Systems, Minneapolis, MN) according to the manufacturer’s protocol.

### Bacterial burden in major organs

For determination of bacterial burden, mice were euthanized on day 1, 3, or 5 after infection (10^4^ CFU), and specific tissues (MLNs, liver, and spleen) were collected. Organs were weighed, hand-mashed, and homogenized with a Stomacher 80-paddle action blender (Seward, Port Saint Lucie, FL). Serial 10-fold dilutions were prepared with sterile PBS supplemented with 2% FBS. A 10-μl volume of each dilution was plated onto LB Strep plates and incubated at 37°C in 5% CO_2_ for 24 hours. Results are expressed as total CFU per tissue.

### Histopathology

Cecum, and colon tissues were fixed and stored in Bouin's solution (VWR Scientific, West Chester, PA). Fixed tissues were embedded in paraffin, sectioned, mounted onto slides, and stained with hematoxylin and eosin. Sections were evaluated in blinded fashion by a single pathologist (Dr. R. T. Bronson, Harvard Medical School); samples were scored from 0 to 4 for degree of observable inflammation.

### Plasmacytoid dendritic cell (pDC) depletion *in vivo*

Diphtheria toxin (200 ng/dose) from *Corynebacterium diphtheriae* (Sigma, St. Louis, MO) was administered to BDCA2-DTR and WT mice four times (days -3, -1, +1, and +3 relative to the day of oral infection with SL1344). Mice were monitored for pDC depletion by measurement of the loss of Siglec H+B220+CD11b-CD11c+ cells in the MLNs and spleen by flow cytometry.

### IL-10R blockade *in vivo*

300 μg/mouse of rat anti-mouse IL-10R mAb (clone: 1B1.3A, rIgG1; BioXcell, West Lebanon, NH) or rat IgG1 isotype control antibody (BioXcell, West Lebanon, NH) was administered intraperitoneally 3 days prior to and on the day of infection with *S*. *typhimurium*. 3 days post infection, an additional dose of 200μg/mouse of the IL-10R mAb or isotype control antibody was administered intraperitoneally.

### Statistical analysis

Statistical analysis was performed using the standard appropriate tests. For survival studies the log-rank test was used to compare differences between the survival of two groups. All other experiments were statistically analyzed with the Mann-Whitney non-parametric test.

## Results

### IFNAR1 signaling contributes to mortality in *S*. *typhimurium*-induced colitis

**Fig 1:**
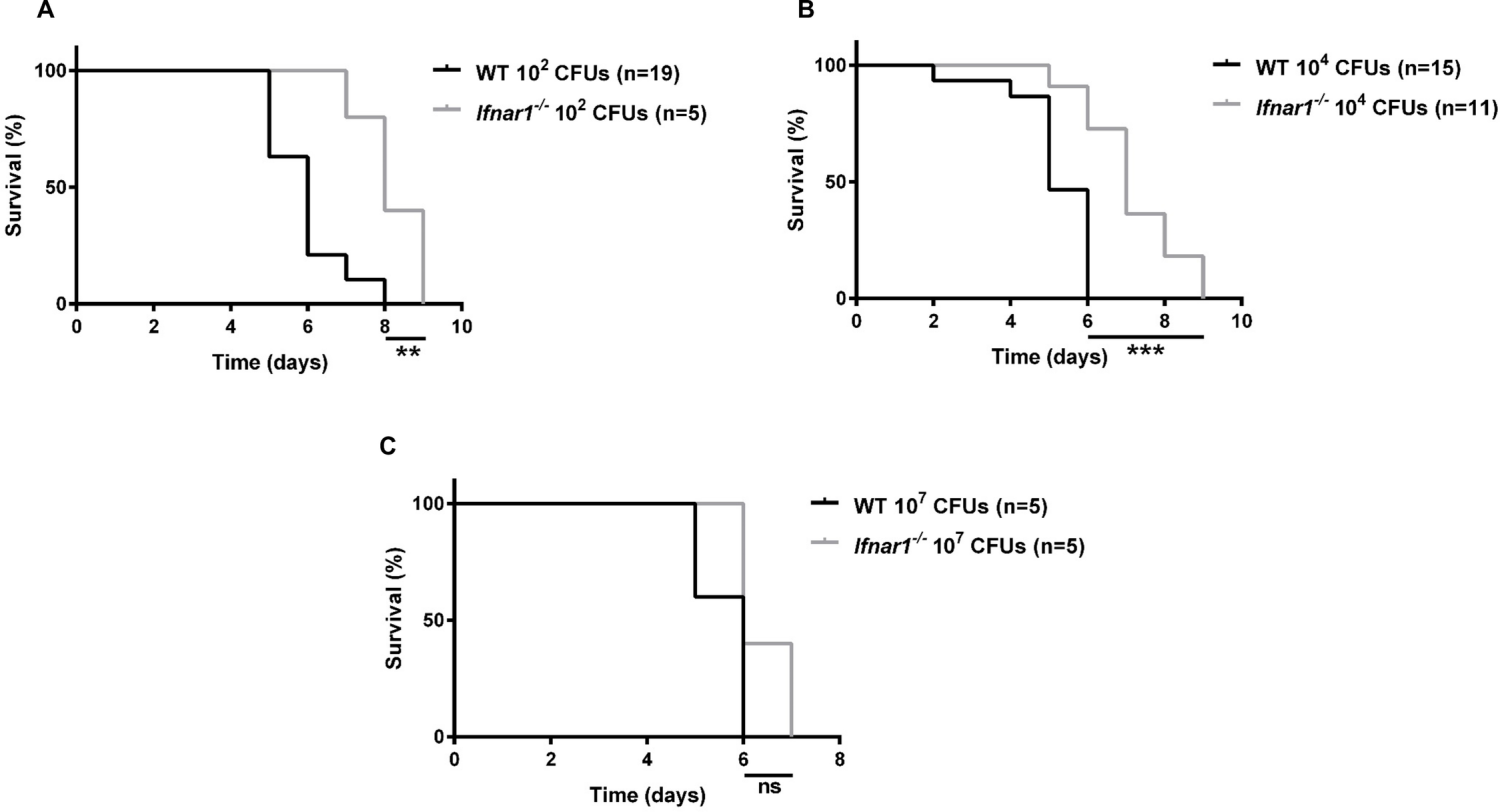
IFNAR1 signaling is pathogenic in *Salmonella* gastroenteritis. *S*. *typhimurium* SL1344 was administered orally to mice at various doses after treatment with streptomycin. Doses: (A) 10^2^ CFU, (B) 10^4^ CFU, (C) 10^7^ CFU. Statistical analysis was performed with a log-rank test. ns, not significant; **p<0.01; ***p<0.001.

To assess the effect of type I IFN signaling on susceptibility to *S*. *typhimurium* infection after streptomycin treatment, we initially investigated the survival of WT and *Ifnar1*^-/-^ animals after infection with various doses of *S*. *typhimurium*. When we used inocula of *S*. *typhimurium* (10^2^ and 10^4^ CFU) that approximate the infectious dose in humans [16], WT mice died significantly faster than *Ifnar1*^*-/-*^ mice (Fig 1A and 1B, respectively). Interestingly, when we used a much larger inoculum (10^7^ CFU), there was no difference in survival between the groups (Fig 1C). This finding suggests that, with a higher infectious burden, another pathway overwhelms the effects of IFNAR1 signaling. These results demonstrate that at lower, clinically relevant doses of *Salmonella*, IFNAR1 signaling is pathogenic in *Salmonella*-induced colitis.

### Colonic and systemic inflammation are augmented by IFNAR1 signaling

**Fig 2:**
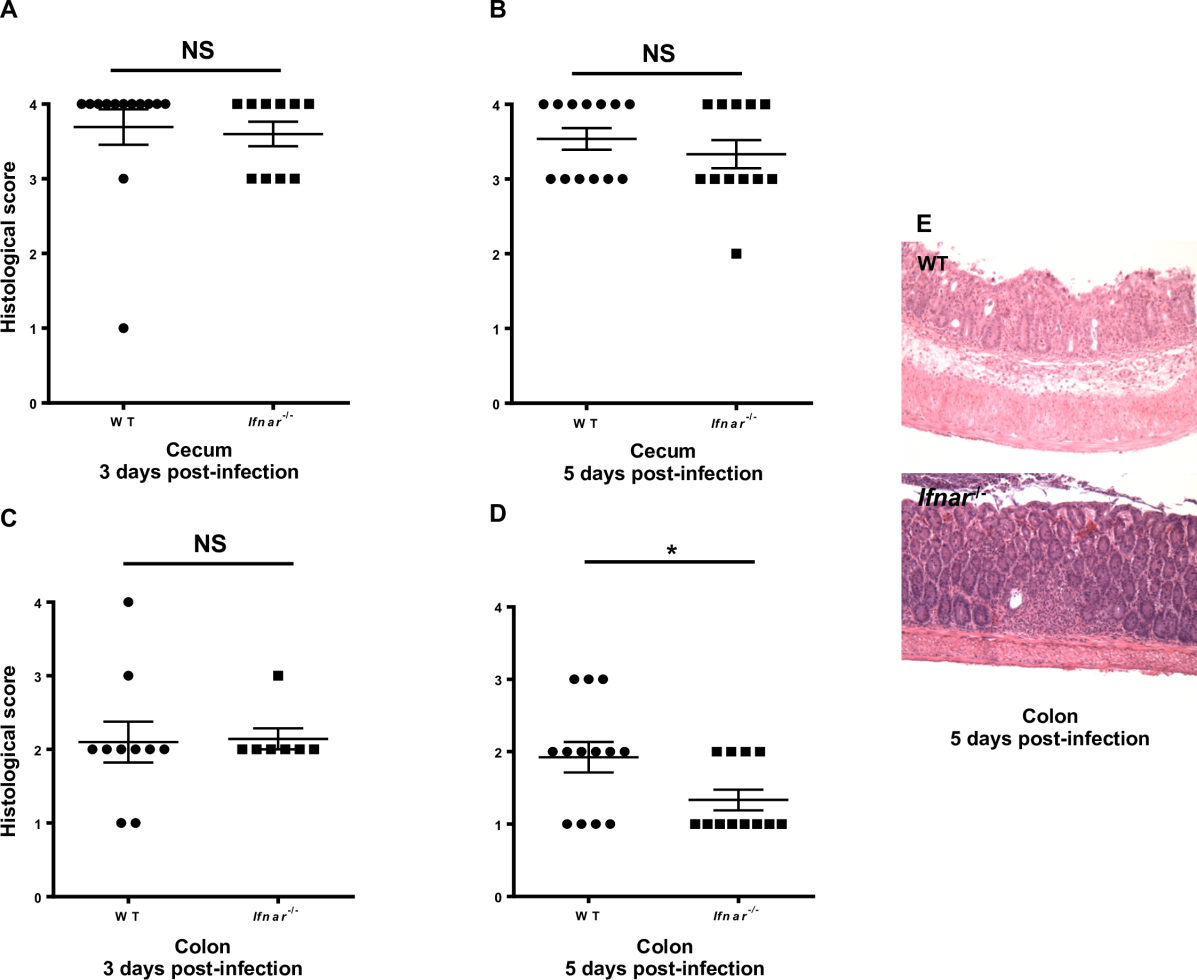
IFNAR1 signaling leads to increased colonic inflammation. Mice were infected by oral gavage with 10^4^ CFU of *S*. *typhimurium* SL1344. (A, B) Inflammation in the cecum on day 3 (A) and day 5 (B) after infection. (C, D) Inflammation in the colon on day 3 (C) and day 5 (D) after infection. (E) Representative images of WT and *Ifnar1*^*-/-*^ colon on day 5 after infection. Villus structure was completely lost and severe edema was observed in WT colon, whereas only minor cellular infiltration was seen in *Ifnar1*^-/-^ mice. Results are mean values ± SEM; statistical analysis was performed with the Mann-Whitney non-parametric test.

To assess the effects of IFNAR1 signaling on *S*. *typhimurium*-induced colonic inflammation [9, 17], we examined and scored the histopathological condition of the colon and cecum of WT and *Ifnar1*^-/-^ mice on days 3 and 5 after infection. The cecum was highly inflamed on both day 3 and day 5, with no difference between WT and *Ifnar1*^-/-^ mice (Fig 2A and 2B, respectively). In contrast, although no difference in colonic inflammation was observed on day 3 (Fig 2C), on day 5 WT mice had significantly greater inflammation (as manifested by edema, immune cell infiltrations, and loss of normal colonic architecture) than did *Ifnar1*^*-/-*^ mice (Fig 2D and 2E).

**Fig 3:**
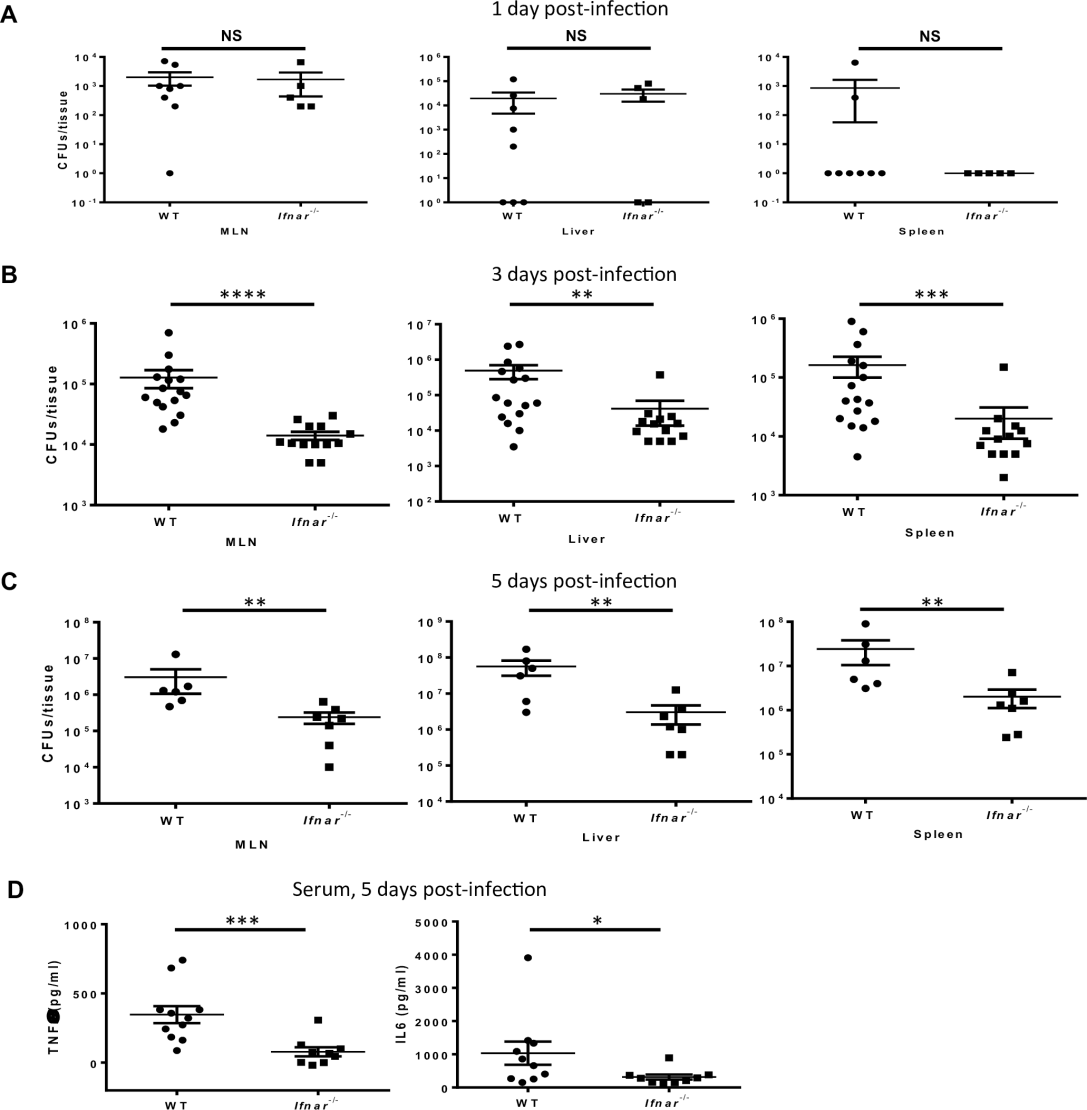
IFNAR1 signaling leads to increased *Salmonella* dissemination. Mice were infected by oral gavage with 10^4^ CFU of *S*. *typhimurium* SL1344. (A-C) *Salmonella* burden in MLNs, liver, and spleen on day 1 (A), day 3 (B), and day 5 (C) after infection. (D) Serum levels of TNF-α and IL-6 in infected animals on day 5 after infection. Results are mean values ± SEM; statistical analysis was performed with the Mann-Whitney test. *p<0.05; **p<0.01; ***p<0.001; ****p<0.0001.

To obtain a better understanding of the dynamics of bacterial dissemination during *Salmonella* gastroenteritis, we examined bacterial burden in several major organs throughout the course of infection. The bacterial burden in the MLNs, liver, and spleen was significantly higher in WT than in *Ifnar1*^*-/-*^ animals on days 3 and 5 after infection (Fig 3B and 3C, respectively). In contrast, bacterial dissemination to these organs was not different on day 1 after infection; thus, these groups of mice had a similar disease burden at an early time point (Fig 3A). To gain insight into the systemic response to infection, we measured serum levels of the pro-inflammatory cytokines TNFα and IL-6 on day 5 after infection. Consistent with the greater degree of bacterial dissemination in WT mice, we found that WT mice had higher serum levels of TNFα and IL-6 than did *Ifnar1*^-/-^ animals (Fig 3D). Overall, these studies demonstrate that WT mice have more severe disease than do *Ifnar1*^*-/-*^ mice, as evidenced by greater colonic inflammation, systemic dissemination of bacteria, and faster death.

To assess whether there were differences in bacterial colonization that were driving the differential outcome of the disease, we measured bacterial CFU in the feces of WT and *Ifnar1*^*-/-*^ mice on day 1 after infection. As in distal organs, where we observed no significant difference in bacterial numbers on day 1 (Fig 3C), we found that bacterial numbers were overall similar in *Ifnar1*^*-/-*^ and WT feces at this early time point (S1 Fig). These results suggest that better overall disease outcome could not be attributed to lower-level initial colonization in *Ifnar1*^*-/-*^ mice.

### pDCs do not play a significant role in pathogenesis during *S*. *typhimurium* infection

**Fig 4:**
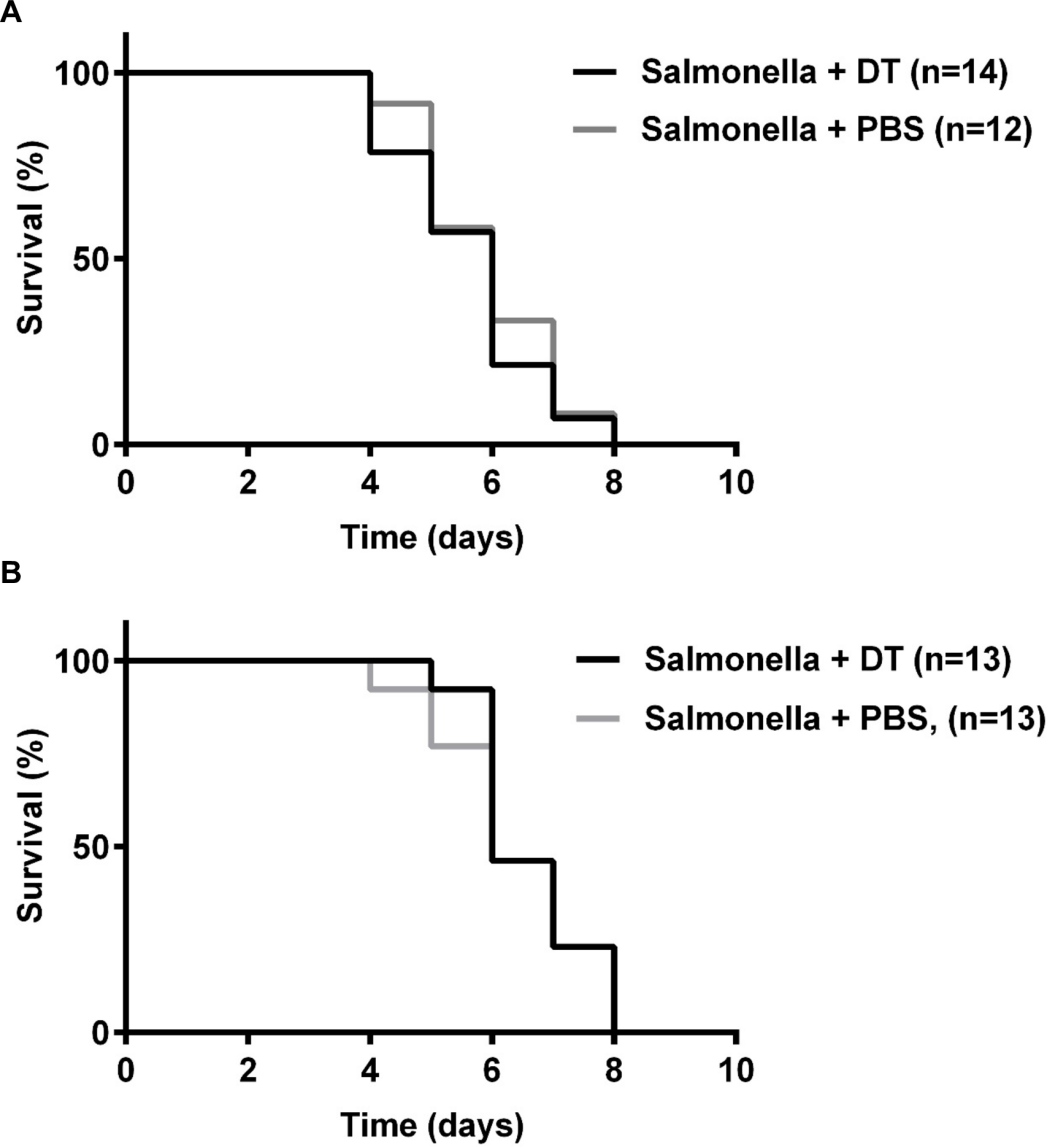
pDCs do not contribute to *Salmonella* pathogenesis. **(**A, B) Survival curves for WT mice (A) and BDCA2-DTR mice (B) given diphtheria toxin (DT) or PBS and infected with 10^4^ CFU of *S*. *typhimurium* SL1344. Statistical analysis was performed with a log-rank test.

Having demonstrated that type I IFN signaling is pathogenic in *Salmonella* infections, we wanted to define which immune cells contribute to this phenotype. pDCs are an important source of type I IFNs and a major initial source of IFNα: their early removal during viral infection delays antiviral immunity while in a murine model of lupus similar removal reduces the disease [2, 18]. In addition, pDCs are involved in the immune response to bacterial pathogens, including both extracellular and intracellular organisms [19–21]. Given their contribution to other viral and bacterial infections and their involvement in other autoimmune and inflammatory diseases, we examined whether pDCs play a role in *S*. *typhimurium* infection. By administering diphtheria toxin (DT) to the well-defined BDCA2-DTR mice, in which pDCs are the only cells that express the diphtheria toxin receptor (DTR), we were able to specifically deplete pDCs. Depletion of pDCs (≥95%) in uninfected mice using this system has previously been demonstrated [15]. We confirmed depletion of pDCs in the spleen and MLN of *Salmonella*-infected mice using flow cytometric analysis (S2 Fig). As a control, we first confirmed that diphtheria toxin administration did not influence mortality in *Salmonella*-infected mice that do not express DTR (Fig 4A). Similarly, depletion of pDCs had no effect on mortality during *S*. *typhimurium* infection (Fig 4B). Thus, the absence of pDCs does not affect the outcome of *Salmonella* colitis.

### *Salmonella*-infected *Ifnar1*^*-/-*^ mice have more CD11c^-^ macrophages and CD103^+^ DCs than WT mice

**Fig 5:**
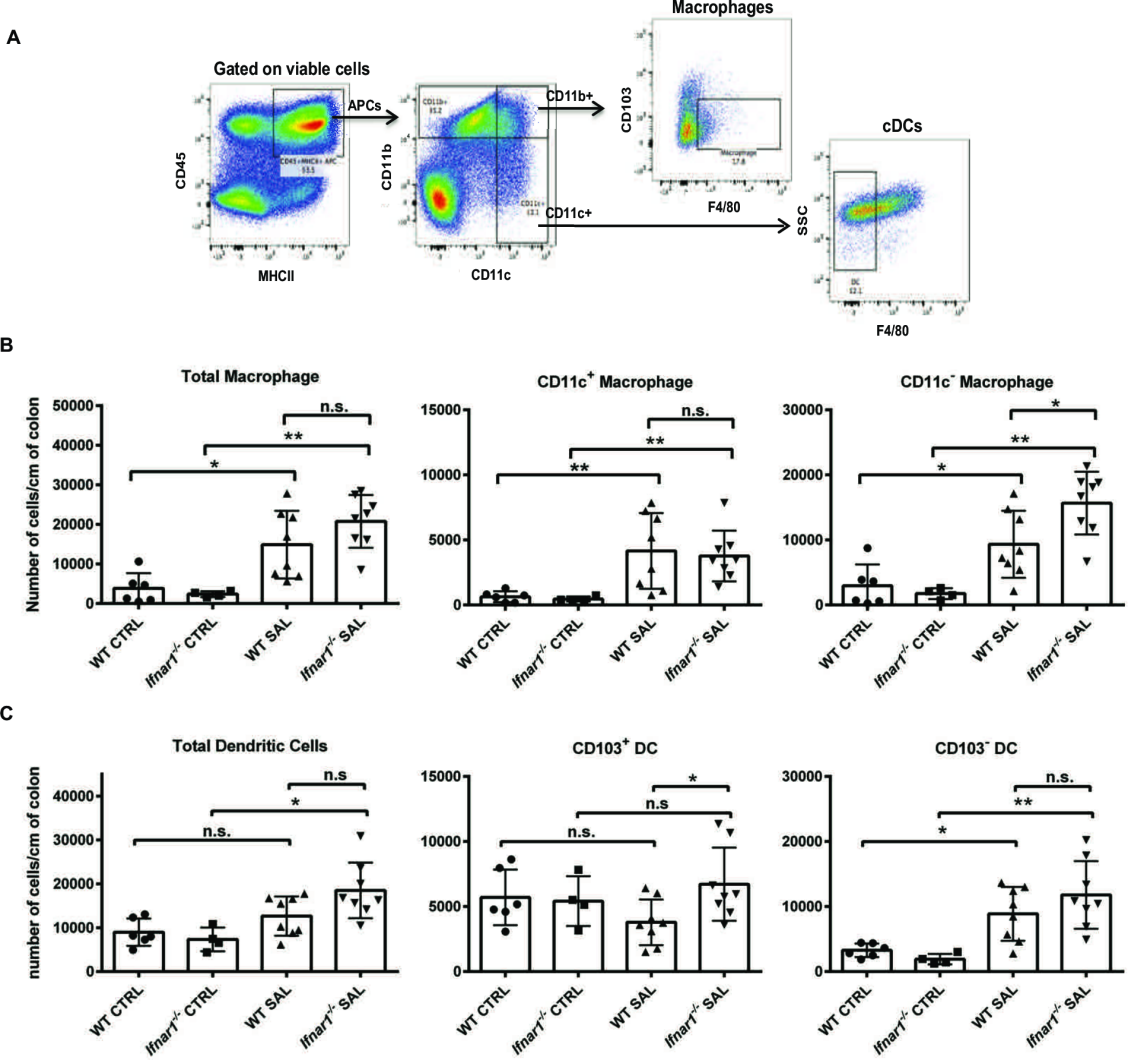
Specific subsets of colonic APCs are modulated by IFNAR1 signaling during *Salmonella* infection. Cells were isolated from the colonic lamina propria of uninfected mice (CTRL) or infected mice on day 3 after oral infection with 10^4^ CFU *Salmonella* (SAL). (A) Gating strategy to identify macrophages and cDCs. (B) Numbers of total, CD11c^+^, and CD11c^-^ colonic macrophages in WT and *Ifnar1*^*-/-*^ mice. (C) Numbers of total, CD103^+^, and CD103^-^ colonic cDCs in WT and *Ifnar1*^*-/-*^ mice. Results are mean values ± SD. Statistical analysis was performed with the Mann-Whitney test. ns, not significant; *p<0.05; **p<0.005.

Since pDCs are dispensable for disease severity, we explored the role of other immune-cell subsets by enumerating innate immune cells in the colon and MLNs on day 3 after infection. We focused our analysis on macrophages (CD45^+^MHCII^+^CD11b^+^F4/80^+^CD103^-^) and conventional DCs (cDCs; CD45^+^MHCII^+^CD11c^+^ F4/80^-^) (Fig 5A), which are major producers of type I IFN [22–24]. Indeed, as indicated previously, macrophages at other sites have been demonstrated to play a major role in *Salmonella* infection [25]. Compared with naïve animals, infected mice had significantly higher total colonic macrophage numbers, with no difference between WT and *Ifnar1*^*-/-*^ mice (Fig 5B, left panel). CD11c expression by colonic macrophages helps distinguish cell subsets that are functionally and anatomically distinct; CD11c^+^ macrophages are present in the lamina propria and CD11c^-^ may be like CD11c^lo^ macrophages in the muscularis mucosa region or draining the tissue during inflammation [13]. While the CD11c^+^ macrophage population did not differ between infected WT and infected *Ifnar1*^*-/-*^ mice, CD11c^-^ macrophages (which represent the majority of colonic macrophages) were more numerous in the colon of *Ifnar1*^-/-^ mice (Fig 5B, center and right panels).

Similar to our findings with colonic macrophages, total numbers of colonic cDCs did not differ between infected WT mice and infected *Ifnar1*^-/-^ mice (Fig 5C). Intestinal cDCs can be distinguished by CD103 expression; CD103^+^ cDCs have important roles in carrying antigens to MLNs and regulating the inflammatory response [14]. Interestingly, there was a marked increase in the colonic CD103^+^ cDC population in *Ifnar1*^*-/-*^ mice over that in WT mice, while the two groups did not differ in numbers of CD103^-^ cDCs (Fig 5C).

Given that the MLN is an important draining lymph node in enteric *Salmonella* infection and is likely to represent the gateway to systemic dissemination, we enumerated macrophages and cDCs in this compartment. The results were quite different from those in the colon. Although the number of macrophages (both CD11c^+^ and CD11c^-^) went up for both WT and *Ifnar1*^*-/-*^ mice in the setting of infection, there was no difference between the two groups (S3 Fig). Moreover, the number of cDCs (total and CD103^+^) in MLNs was higher in infected WT animals than in infected *Ifnar1*^*-/-*^ mice (S3 Fig).

### Increase in tolerogenic IL-10R^+^ colonic antigen-presenting cells (APCs) in the absence of IFNAR1 signaling

**Fig 6:**
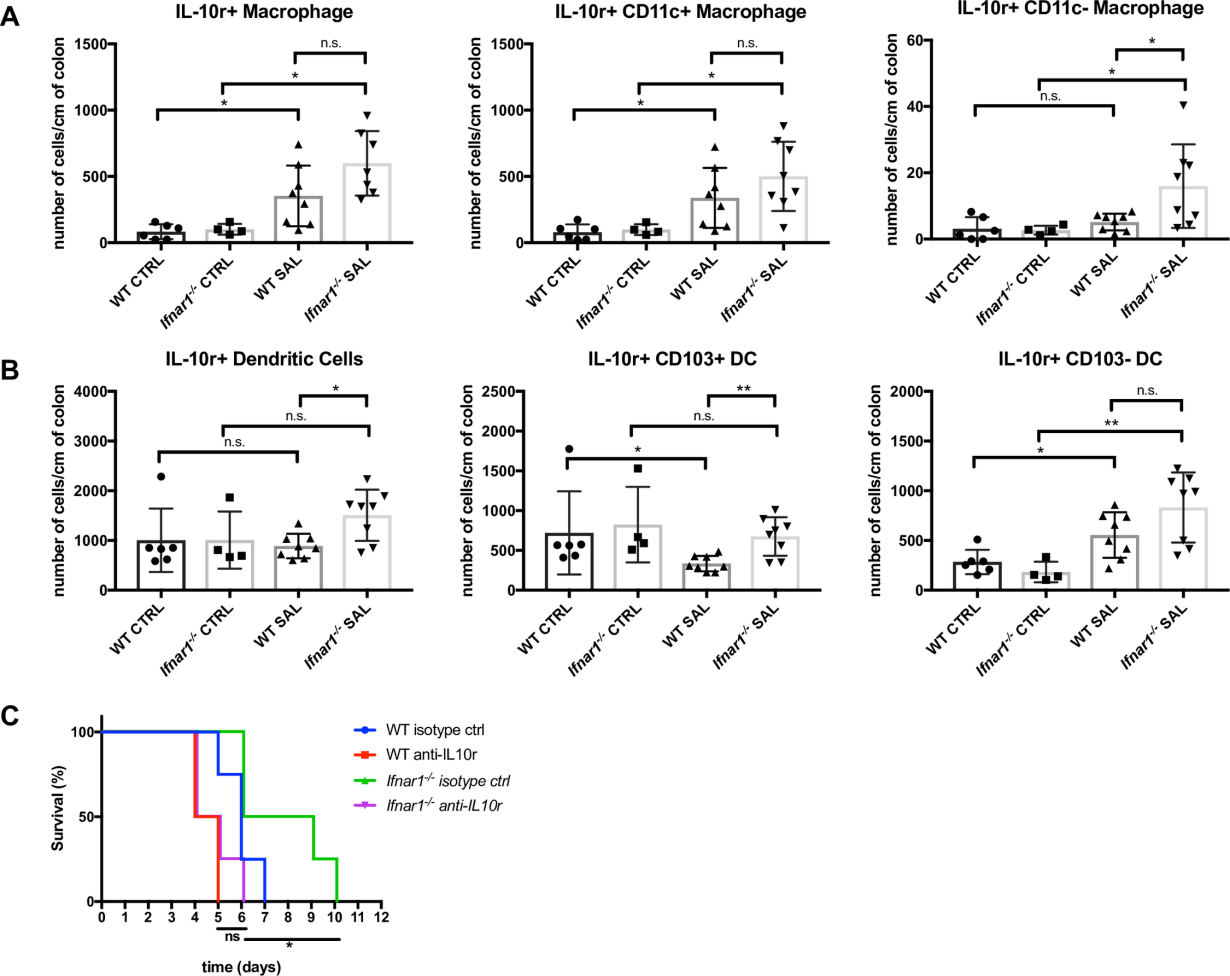
IFNAR1 signaling is associated with a decrease in IL-10R^+^ colonic APCs in *Salmonella*-infected mice. (A-B) Cells were isolated from the colonic lamina propria of uninfected mice (CTRL) or on day 3 post-infection from mice orally infected with 10^4^ CFU *Salmonella* (SAL). (A) The number of IL-10R^+^ colonic macrophages, CD11c^+^ IL-10R^+^, and CD11c^-^ IL-10R^+^ macrophages were enumerated in WT and *Ifnar1*^*-/-*^ mice. (B) The number of total IL-10R^+^ cDCs, CD103^+^ IL-10R^+^ cDCs, and CD103^-^ IL-10R^+^ cDCs were enumerated in WT and *Ifnar1*^*-/-*^ mice. Results represent the mean with SD. Statistical analysis was performed using the Mann-Whitney test. *p<0.05, **p<0.005. (C) Survival curve of WT and *Ifnar1*^*-/-*^ mice treated with anti-IL-10R mAb (anti-IL-10R) or isotype control antibody (isotype) on days -3, 0, and +3 and infected with 10^4^ CFU *Salmonella*. Statistical analysis was performed using a log-rank test. ns, not significant; *p<0.05.

Colonic macrophages expressing the receptor of anti-inflammatory cytokine IL-10 (IL-10R) have anti-inflammatory properties and protect against colitis [26, 27]. Given that numbers of specific macrophage and dendritic cell subsets were increased in *Ifnar1*^*-/-*^ mice, which have less severe disease than WT animals, we investigated whether these cell types correspond to tolerogenic subsets marked by IL-10R. Indeed, while the number of IL-10R^+^ CD11c^+^ macrophages increased similarly in infected WT and *Ifnar1*^*-/-*^ mice, *Salmonella*-infected *Ifnar1*^*-/-*^ mice had more IL-10R^+^ CD11c^-^ macrophages than did infected WT mice (Fig 6A). Similarly, *Salmonella*-infected *Ifnar1*^*-/-*^ mice had more IL-10R^+^ CD103^+^ cDCs than infected WT mice because of a decrease in this cell type in the setting of type I IFN signaling (Fig 6B).

### Blockade of IL-10R increases susceptibility of *Ifnar1-/-* mice to *S*. *typhimurium*

Based on the observed increase in IL-10R expressing CD11c^-^ macrophages and CD103^+^ cDCs in *S*. *typhimurium* infected *Ifnar1*^*-/-*^ mice compared to WT mice, we sought to investigate the role of IL-10R in the reduced susceptibility of these mice to *S*. *typhimurium* colitis. Blockade of IL-10R in *Ifnar1*^*-/-*^ mice significantly reduced the time to death of these mice compared to isotype control treated *Ifnar1*^*-/-*^ mice (Fig 6C). Notably, with blockade of IL-10R, the difference in susceptibility of WT and *Ifnar1*^*-/-*^ mice is no longer observed (Fig 6C). Taken together, these data suggest that type I IFN signaling may lead to more severe disease in *Salmonella* infections by downregulating these immunoregulatory, IL-10R expressing APC subsets.

## Discussion

Infection with *S*. *typhimurium* elicits a strong inflammatory response that benefits the bacteria by creating an environment that provides a growth advantage for *Salmonella* over members of the microbiota [11]. Therefore, the ability of the host to actively limit an excessive inflammatory response can be advantageous to the host even in the context of bacterial infection. Here, we demonstrate that type I IFN signaling is pathogenic in the murine model of *Salmonella* gastroenteritis, as evidenced by shorter time to death, greater bacterial dissemination, higher serum levels of pro-inflammatory cytokines, and greater colonic inflammation.

In light of the differences in the degree of colonic inflammation between WT and *Ifnar1*^*-/-*^ animals, we characterized prominent colonic APCs, focusing on cell types known to produce type I IFN. We found that numbers of CD11c^-^ macrophages but not CD11c^+^ macrophages differed in the two groups, with higher counts in *Ifnar1*^*-/-*^ mice. Since these macrophage subsets have different anatomic localizations and distinct functional characteristics [13], CD11c^-^ macrophages whose numbers increase in the absence of IFNAR1 signaling may possibly contribute to the tolerogenic phenotype observed in *Ifnar1*^*-/-*^ mice. Interestingly, *Ifnar1*^*-/-*^ mice also had an increased number of CD11c^-^ macrophages expressing IL-10R, a recently described immunoregulatory subset of macrophage. IL-10R^+^ colonic macrophages are crucial in preventing colitis [26, 27], and IL-10R mutations in humans are associated with the early onset of inflammatory bowel disease [28]. In addition, lack of IL10R mediated signaling has recently been demonstrated to change macrophage programming to an activated inflammasome pathway driven by mTORC1 [29]. Further investigation will be required to determine whether blocking IL10R in *Ifnar1*^*-/-*^ mice causes earlier death mediated by mTORC1/inflammasome activation. Taken together, these findings suggest that IL-10R^+^ macrophages are important for maintenance of colonic homeostasis and prevention of excessive intestinal inflammation.

Our results demonstrate that IFNAR1 signaling results in a decreased number of IL-10R^+^ macrophages, although the mechanism by which these cell numbers are modulated is unclear. One possibility is that signaling by type I IFNs leads directly to a reduction of these immunoregulatory macrophages. Given recent data demonstrating that IL-10 levels after intraperitoneal infection with *Salmonella* are diminished in the absence of IFNβ [12], an alternative, mutually non-exclusive possibility is that IL-10 levels are decreased in *Salmonella*-infected *Ifnar1*^*-/-*^ mice with a compensatory increase in the expression of IL-10R. Zigmond et al. found that IL-10R on macrophages- not IL-10 secreted by macrophages- was critical for the anti-inflammatory properties of this macrophage subset [27], suggesting that it is the altered number of IL-10R macrophages that matters, irrespective of the underlying mechanism.

Although pDCs are involved in regulating immunity to viral and some bacterial infections, we found that they do not contribute significantly to the pathogenesis of *Salmonella* infection. Rather, we found that numbers of colonic CD103^+^ cDCs, which are known to be important for uptake and presentation of bacterial antigens during *S*. *typhimurium* infection [30, 31], were lower in WT mice than in *Ifnar1*^*-/-*^ mice. Moreover, CD103^+^ IL-10R^+^ cDC numbers were lower in infected WT mice than in uninfected mice, with no change in *Ifnar1*^*-/-*^ mice. These results suggest that *Salmonella*-induced IFNAR1 signaling leads to the decrease in CD103^+^ IL-10R^+^ cDCs. While IL-10R expression in macrophages is crucial for immune tolerance, the connection between IL-10R expression and DC function has not yet been fully addressed. On the basis of our demonstration of an association between a numerical decrease in these cells and worse outcomes, it is intriguing to speculate that IL-10R^+^ cDCs are similarly involved in immunoregulation.

In summary, we have demonstrated that type I interferon signaling is detrimental to the host in a model of *Salmonella*-induced colitis. Furthermore, CD11c^-^ IL-10R^+^ colonic macrophages are more abundant in *Ifnar1*^*-/-*^ mice than in WT mice, and blockade of IL-10R eliminated the mortality differences between WT and *Ifnar1*^*-/-*^ mice. These results suggest that these macrophages may not only play a role in maintaining intestinal homeostasis but may also have anti-inflammatory functions during severe bacterial infection. Future investigation of how these different signaling pathways interact with each other during invasion by a mucosal pathogen would lead to a better understanding of immune defense and might contribute to the design of novel approaches to mucosal vaccination for infectious diseases of the GI tract.

## Supporting information

S1 Fig Initial colonization of WT and *Ifnar1*^*-/-*^ mice by *S*. *typhimurium* preceding antibiotic treatment. Mice were infected by oral gavage with 10^4^ CFU of *S*. *typhimurium* SL1344; bacterial burden was assessed in the feces on day 1 after infection. Results are mean values ± SEM. Statistical analysis was performed with the Mann-Whitney non-parametric test. *p<0.05.
(TIF)

S2 Fig Depletion of pDCs in *Salmonella-*infected BDCA2-DTR mice. BDCA2-DTR mice were administered 200ng diphtheria toxin (+DT) or PBS (-DT) on days -3, -1, +1, and +3 with respect to the day of *Salmonella* infection to deplete pDCs. On day 0, mice were infected with 10^7^ CFU *S*. *typhimurium* SL1344 by oral gavage. pDC depletion was monitored in the MLN (A) and spleen (B) using flow cytometry to measure the loss of SiglecH+B220+CD11b-CD11c+ cells. Results represent the mean of the total number of pDCs per sample ± SD. Statistical analysis was performed using the Mann-Whitney non-parametric test. *p<0.05.
(TIF)

S3 Fig Specific subsets of APCs in the MLNs are modulated by IFNAR1 signaling during *Salmonella* infection. Cells were isolated from MLNs of uninfected mice (CTRL) and from MLNs of infected mice on day 3 after infection with 10^4^ CFU of *Salmonella* (SAL). (A) Numbers of total, CD11c^+^, and CD11c^-^ macrophages in MLNs from WT and *Ifnar1*^*-/-*^ mice. (B) Numbers of total, CD103^+^, and CD103^-^ cDCs in MLNs from WT and *Ifnar1*^*-/-*^ mice. Results are mean values ± SD. Statistical analysis was performed with the Mann-Whitney test. *p<0.05; **p<0.005.
(TIF)
