# Supplementary figures and images for: Type I interferon signaling restrains IL-10R+ colonic macrophages and dendritic cells and leads to more severe Salmonella colitis
Source: PLoS One. 2017 Nov 30;12(11):e0188600. doi: 10.1371/journal.pone.0188600 (PMC5708670; doi:10.1371/journal.pone.0188600)

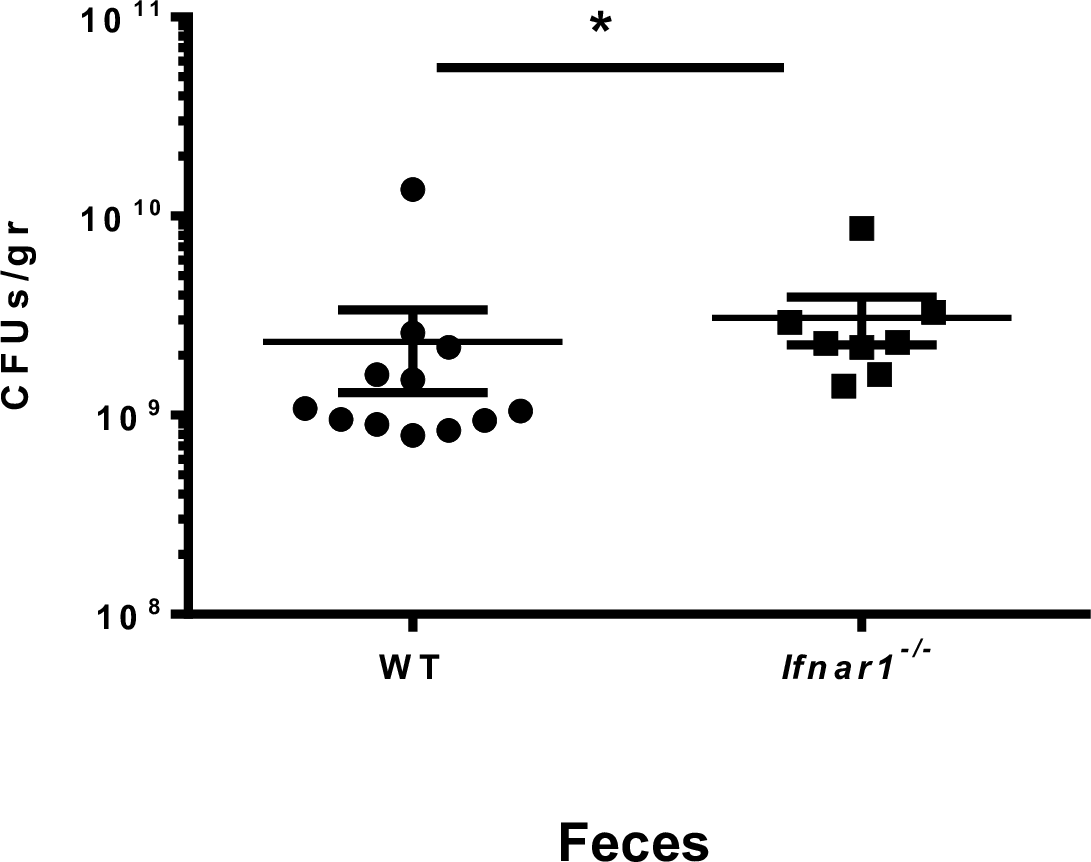

Supplement: S1 Fig — Mice were infected by oral gavage with 104 CFU of S. typhimurium SL1344; bacterial burden was assessed in the feces on day 1 after infection. Results are mean values ± SEM. Statistical analysis was performed with the Mann-Whitney non-parametric test. *p<0.05. (TIF) [file pone.0188600.s001.tif]

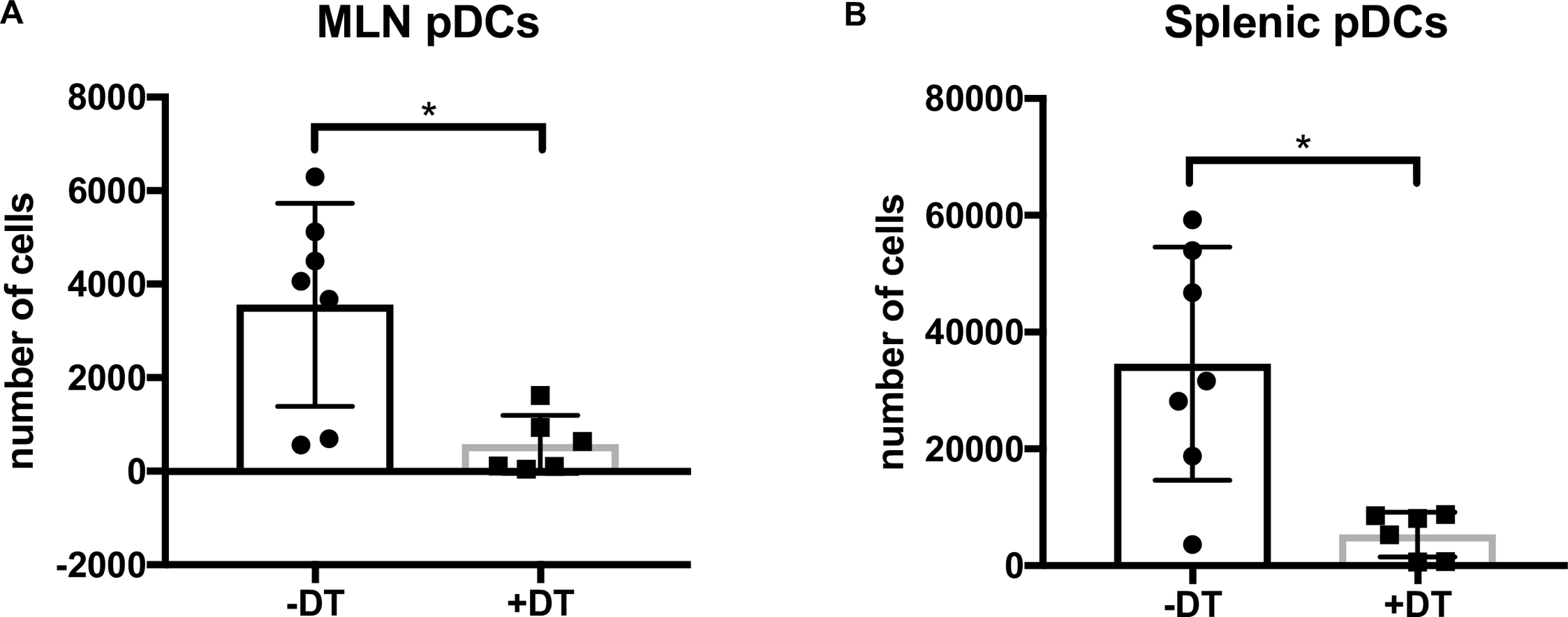

Supplement: S2 Fig — BDCA2-DTR mice were administered 200ng diphtheria toxin (+DT) or PBS (-DT) on days -3, -1, +1, and +3 with respect to the day of Salmonella infection to deplete pDCs. On day 0, mice were infected with 107 CFU S. typhimurium SL1344 by oral gavage. pDC depletion was monitored in the MLN (A) and spleen (B) using flow cytometry to measure the loss of SiglecH+B220+CD11b-CD11c+ cells. Results represent the mean of the total number of pDCs per sample ± SD. Statistical analysis was performed using the Mann-Whitney non-parametric test. *p<0.05. (TIF) [file pone.0188600.s002.tif]

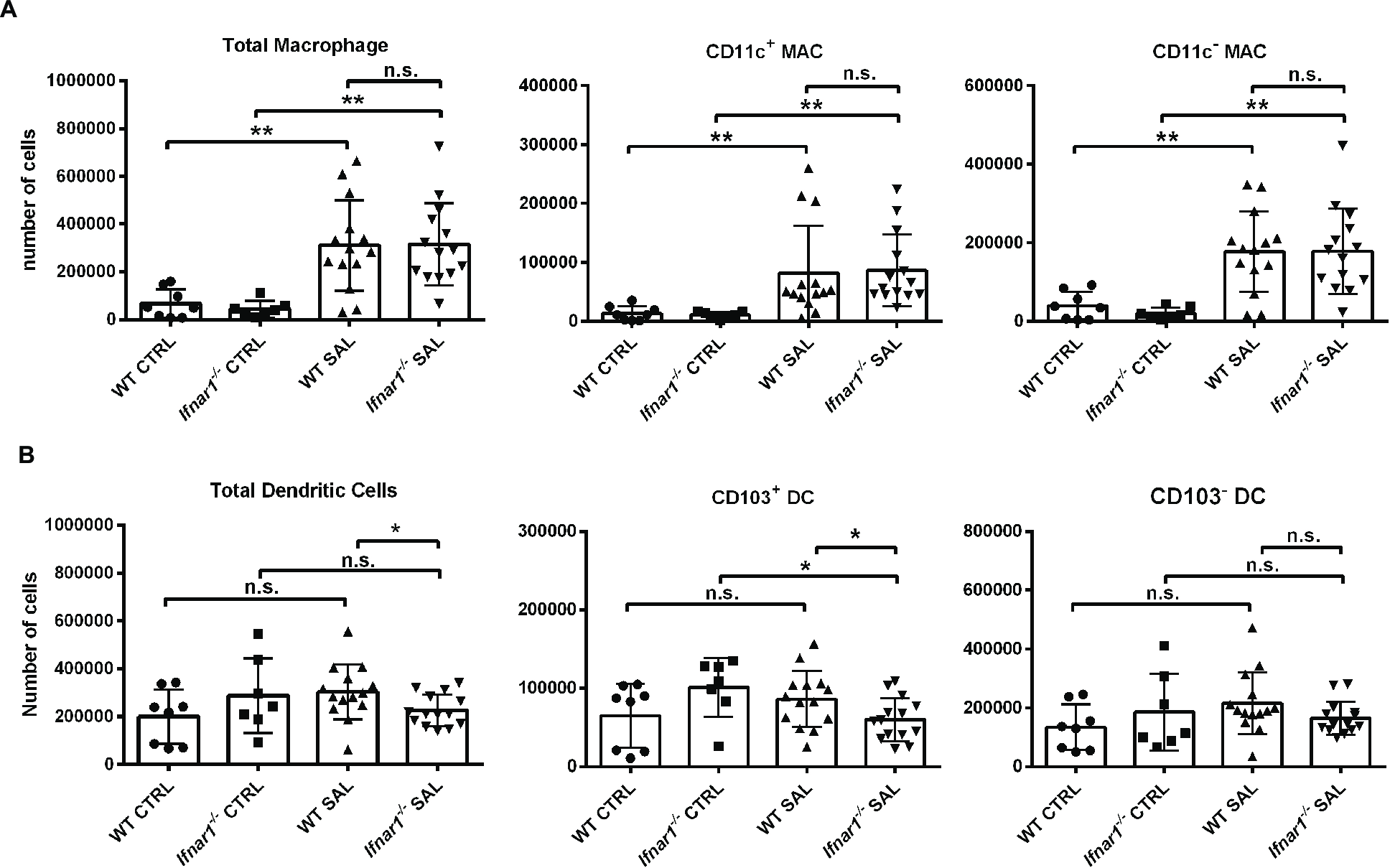

Supplement: S3 Fig — Cells were isolated from MLNs of uninfected mice (CTRL) and from MLNs of infected mice on day 3 after infection with 104 CFU of Salmonella (SAL). (A) Numbers of total, CD11c+, and CD11c- macrophages in MLNs from WT and Ifnar1-/- mice. (B) Numbers of total, CD103+, and CD103- cDCs in MLNs from WT and Ifnar1-/- mice. Results are mean values ± SD. Statistical analysis was performed with the Mann-Whitney test. *p<0.05; **p<0.005. (TIF) [file pone.0188600.s003.tif]
